# T cell signaling and Treg dysfunction correlate to disease kinetics in IL-2Rα-KO autoimmune mice

**DOI:** 10.1038/s41598-020-78975-y

**Published:** 2020-12-15

**Authors:** Genevieve N. Mullins, Kristen M. Valentine, Mufadhal Al-Kuhlani, Dan Davini, Kirk D. C. Jensen, Katrina K. Hoyer

**Affiliations:** 1 Quantitative and Systems Biology Graduate Program, University of California Merced, Merced, CA 95343 USA; 2 Department of Molecular and Cell Biology, School of Natural Sciences, University of California Merced, Merced, CA 95343 USA; 3 Health Sciences Research Institute, University of California Merced, Merced, CA 95343 USA

**Keywords:** Adaptive immunity, Autoimmunity, Cytokines, Signal transduction

## Abstract

IL-2Rα, in part, comprises the high affinity receptor for IL-2, a cytokine important in immune proliferation, activation, and regulation. IL-2Rα deficient mice (IL-2Rα-KO) develop systemic autoimmune disease and die from severe anemia between 18 and 80 days of age. These mice develop kinetically distinct autoimmune progression, with approximately a quarter dying by 21 days of age and half dying after 30 days. This research aims to define immune parameters and cytokine signaling that distinguish cohorts of IL-2Rα-KO mice that develop early- versus late-stage autoimmune disease. To investigate these differences, we evaluated complete blood counts (CBC), antibody binding of RBCs, T cell numbers and activation, hematopoietic progenitor changes, and signaling kinetics, during autoimmune hemolytic anemia (AIHA) and bone marrow failure. We identified several alterations that, when combined, correlate to disease kinetics. Early onset disease correlates with anti-RBC antibodies, lower hematocrit, and reduced IL-7 signaling. CD8 regulatory T cells (Tregs) have enhanced apoptosis in early disease. Further, early and late end stage disease, while largely similar, had several differences suggesting distinct mechanisms drive autoimmune disease kinetics. Therefore, IL-2Rα-KO disease pathology rates, driven by T cell signaling, promote effector T cell activation and expansion and Treg dysfunction.

## Introduction

IL-2 is a cytokine important for proliferation of activated T cells, and survival and function of Tregs^1–4^. The receptor for IL-2 is comprised of three chains—IL-2Rα (CD25), IL-2Rβ (CD122), and the common gamma chain (γ_c_; CD132)^1,5^. IL-2 binding to its receptor activates three signaling pathways, MAPK, PI3K, and JAK1/3-STAT5 pathways^4–6^. These pathways promote cell survival and proliferation, and STAT5-mediated gene expression. IL-2 signaling is strongest and most stable when IL-2Rα is present^1,5^. IL-15, a common-gamma cytokine, also utilizes the same β and γ receptor chains, while IL-7 shares the γ chain^6,7^. Mice deficient in IL-2 (IL-2-KO) or its receptor (IL-2Rα-KO or IL-2Rβ-KO) develop spontaneous, multi-organ autoimmune disease, including autoimmune hemolytic anemia (AIHA), bone marrow (BM) failure (BMF), inflammatory bowel disease, and dacryoadenitis^3,8–13^. Decreased IL-2 signaling reduces survival and suppressive function of regulatory T cells (Tregs) that primarily drives disease^1,3,14,15^.

Signaling through IL-2 lowers the threshold of TCR signaling required to initiate proliferation in CD8 T cells, but not in CD4 T cells^16^. In CD8 T cells, IL-2 signaling strength is important in memory development, with lower signaling strength associated with central memory development rather than effector memory^7,17^. Due to the shared IL-2Rβ subunit, these differentiation signals occur in response to both IL-2 and IL-15^7^. In CD4 Tregs, IL-2 signaling stabilizes STAT5-mediated gene expression of FoxP3 promoting suppressive function^5,14^.

We set out to evaluate the mechanisms underlying differences in disease kinetics in IL-2Rα-KO mice. IL-2Rα-KO mice develop severe autoimmune disease at different rates with some succumbing to disease rapidly and others progressing more slowly^10^. AIHA, thought to be the primary cause of death in IL-2 and IL-2Rα-KO mice is caused by autoantibodies against RBC antigens and is measured by the frequency of antibodies bound to RBCs in combination with anemia severity. RBC life span is thus reduced by Fc-mediated phagocytosis and complement-mediated lysis. Conventional AIHA therapy includes splenectomy and general immunosuppressive and anti-inflammatory regimens. As IL-2Rα-KO T cells do not proliferate in response to low-dose IL-2^10^, but may still signal with high IL-2 availability, we explored potential differences in IL-2 signaling cascades affecting disease progression. At day 19, we found that symptoms associated with AIHA were more severe in mice that develop early disease versus late disease, and the frequency of common lymphoid progenitors (CLP) and peripheral CD4 and CD8 T cells was increased in early disease. TCR signaling kinetics in the lymph node differed between early and late disease mice and T cells from early disease were more apoptotic. IL-2Rα-KO CD8 T cells from both early and late disease mice responded normally to IL-2, but IL-7 and IL-15 responses differed between early and late disease IL-2Rα-KO T cells. Alterations in signaling responsiveness may drive changes in Treg suppression and T cell differentiation, skewing disease kinetics.

## Results

### Early RBC characteristics predict disease kinetics

**Figure 1:**
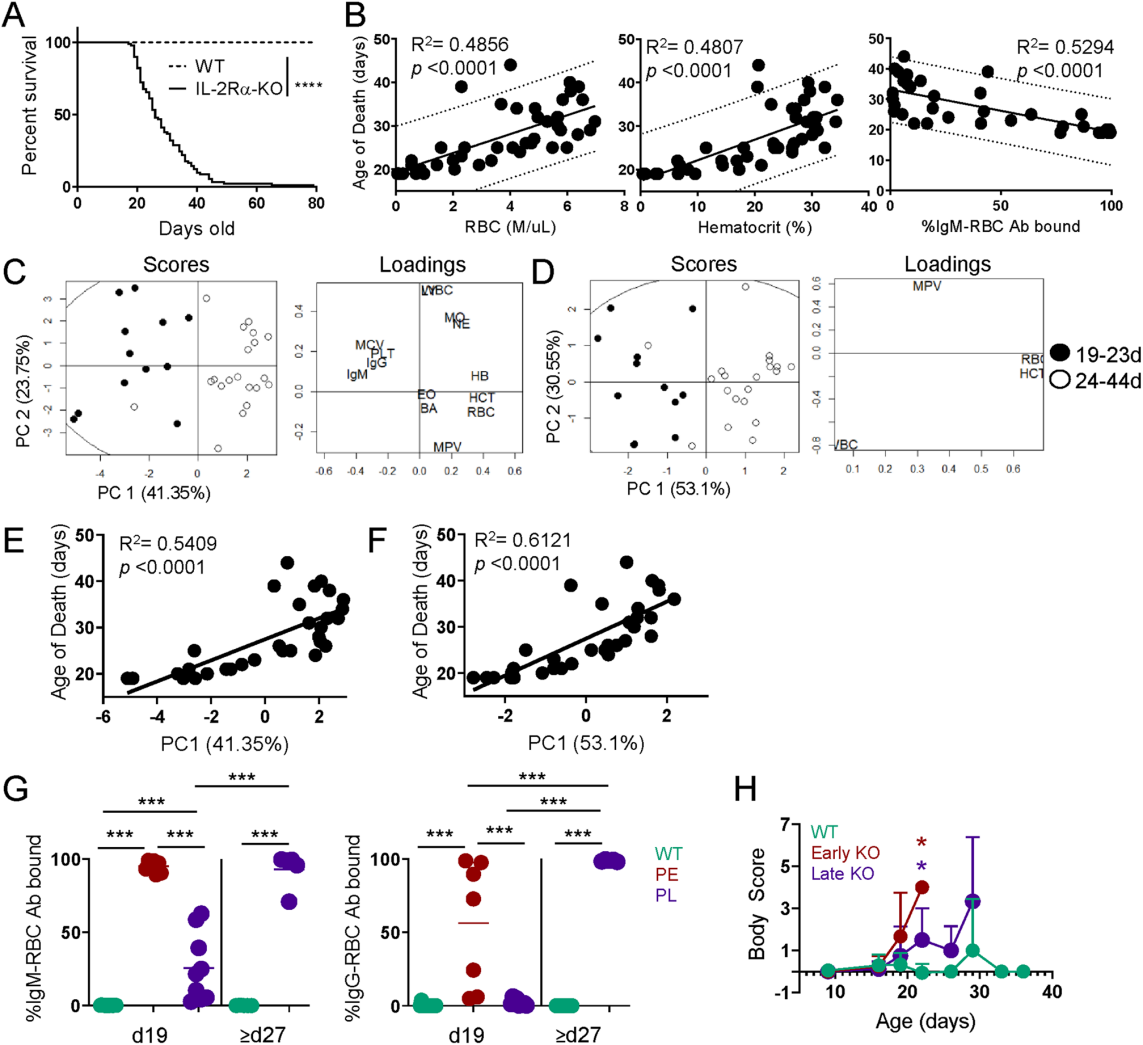
Early RBC characteristics predict IL-2Rα-KO disease kinetics. (**A**) Kaplan–Meier survival curves for WT and IL-2Rα-KO mice. Statistics: log-rank (Mantel–Cox) test, ***p < 0.001; *n* = 90 IL-2Rα-KO and 47 WT mice. (**B**) Dot plots and linear correlations for data collected from day 19 CBC and RBC Ab binding were tested against the age of death of IL-2Rα-KO mice. Pearson correlations with the highest R^2^ values are shown. Dotted lines indicate the 95% prediction interval. Statistics: *p* values shown on graphs indicate whether the slope of the linear regression is significantly non-zero; *n* = 44 (RBC and HCT), *n* = 36 (IgM). (**C**,**E**) PCA by singular value decomposition was performed on peripheral blood collected from 19-day old IL-2Rα-KO using 14 variables or (**D**,**F**) 4 variables. Percentage on the axis indicate the amount of variance in the data that the principal component explains. (**E**,**F**) PC1 is shown against the age of death of the mice. Pearson R^2^ values are shown. Statistics: *p* values shown on graphs indicate whether the slope of the linear regression is significantly non-zero. *n* = 32 mice. (**G**) The frequency of RBC bound by IgM or IgG antibodies at day 19 and late endpoint (d27+). *n* = 7–15 mice per group for day 19, *n* = 5 mice per group for day 27 or older. Statistics: one-way ANOVA with multiple comparisons ***p < 0.001. (**H**) Body score of IL-2KO and WT mice are shown from day 9 onward. *n* = 4 (PE), 11 (PL), 36 (WT) mice. Statistics: multiple t tests with Bejamini–Hochberg FDR correction; *p < 0.001. Standard CBC abbreviations are used^67^.

To assess general survival and disease progression in IL-2Rα-KO mice, survival was monitored. IL-2Rα-KO mice die due to anemia-induced hypoxia from 18 to 79 days, with a median survival of 26 days (Fig. 1A). Twenty-six percent of IL-2Rα-KO mice die between 19 and 21 days of age and 38% die after 30 days. Given the rapid disease in IL-2-KO mice, where 100% of mice die by 25 days in our colony, this larger window of disease kinetics was surprising^18,19^. Separation of mice into distinct early and late disease categories based on the age of death may allow an evaluation of early disease drivers.

To assess disease progression in relation to disease outcome, we first sought a biomarker to predict IL-2Rα-KO age of death. Peripheral blood was collected from IL-2Rα-KO and WT mice at 19 days and evaluated by CBC and bound red blood cell (RBC) antibodies. Mice were monitored for survival, and the age of death was recorded and correlated to the parameters from CBC and RBC antibody detection. The strongest Pearson correlations were RBC concentration (R^2^ = 0.4856), hematocrit (R^2^ = 0.4807), and percentage RBC bound by IgM antibodies (R^2^ = 0.5294) (Fig. 1B). While these showed clear correlations, the 95% prediction interval was ± 10 days, indicating each variable alone does not allow for accurate predictions.

Linear correlation tests alone were not strong enough to predict the age of death but did suggest that blood parameter measurements were strongly correlated with disease outcome; we therefore sought to improve the prediction strength by combining variables together. PCA was performed on variables from CBC and RBC antibody detection. Initial PCA performed on a total of 14 blood variables separated 96.9% of mice into two groups by age of death with 65.1% of variance explained within the first two principal components (Fig. 1C). Regression tree analysis on these data revealed four variables—RBC concentration, hematocrit, white blood cell numbers, and mean platelet volume—as being most important in separating mice by age of death. PCA performed using these four variables split IL-2Rα-KO mice into two groups by age of death, a group of mice that die early (between 19 and 24 days) and a group of mice that die late (24 days or older) with 83.65% of variance explained within the first two principal components (Fig. 1D). Using the four blood parameters, the death could be predicted in 93.8% of mice at 19 days. Furthermore, PC1 in the 4-variable PCA was more highly correlated to the age of death than the 14-variable PC1 (Fig. 1E,F). Together our results reveal a diagnostic tool to predict disease outcome in IL-2Rα-KO mice using blood samples collected early in disease progression.

To assess AIHA disease in relation to disease kinetics, IL-2Rα-KO mice at day 19 were categorized into predicted early (PE) with rapid disease progression or predicted late (PL) with delayed disease progression groups using the four-parameter PCA analysis. Endpoints in the middle time frame (22–26 days) had higher variability, thus a more stringent breakdown of PE (19–21 days) and PL (≥ 27 days) disease groups was utilized for subsequent disease analysis. RBCs collected from these mice were evaluated for binding of IgM and IgG antibodies to evaluate warm (IgG) and cold (IgM) AIHA, each with unique and overlapping etiology, although warm AIHA tends to induce more severe hemolysis^20,21^. Mice in the PE disease group have significantly more RBCs bound by IgM and IgG antibodies than PL mice at day 19 (Fig. 1G). However, PL mice eventually develop high RBC frequencies bound by antibody at disease endpoint (Fig. 1G). At disease endpoint for both groups, day 19–21 PE mice and day 27+ late disease mice show similar signs of advanced anemia, including pale extremities, hunched appearance, and dyspnea (Fig. 1H, Supplementary Table S1). This indicates that early AIHA development leads to early death in IL-2Rα-KO mice, and that several blood parameters, including antibody-binding to RBCs, can be highly predictive of early disease onset.

Given that class-switched IgG antibodies bind to RBCs, we assessed whether this was concomitant to a difference in follicular T cells that mediate B cell class-switching^19,22^. We found that splenic CD4 T follicular helper and CD8 T follicular cell populations were significantly increased in IL-2Rα-KO mice (Supplementary Fig. S1A). CD8 Tfc was also mildly increased in PE compared to PL groups, but the difference didn’t reach significance. These observations may account for the difference in frequency of anti-RBC IgG at the same timepoint (Fig. 1G), although a direct comparison between T follicular cells and RBC antibodies did not correlate. By disease endpoint, PL IL-2Rα-KO mice also exhibited significantly increased CD8 Tfc and CD4 Tfh frequency and total number, contributing to endpoint levels of anti-RBC IgG (Supplementary Fig. S1A,B).

### Altered T cell ratio and activation/memory profiles in IL-2Ra-KO mice

**Figure 2:**
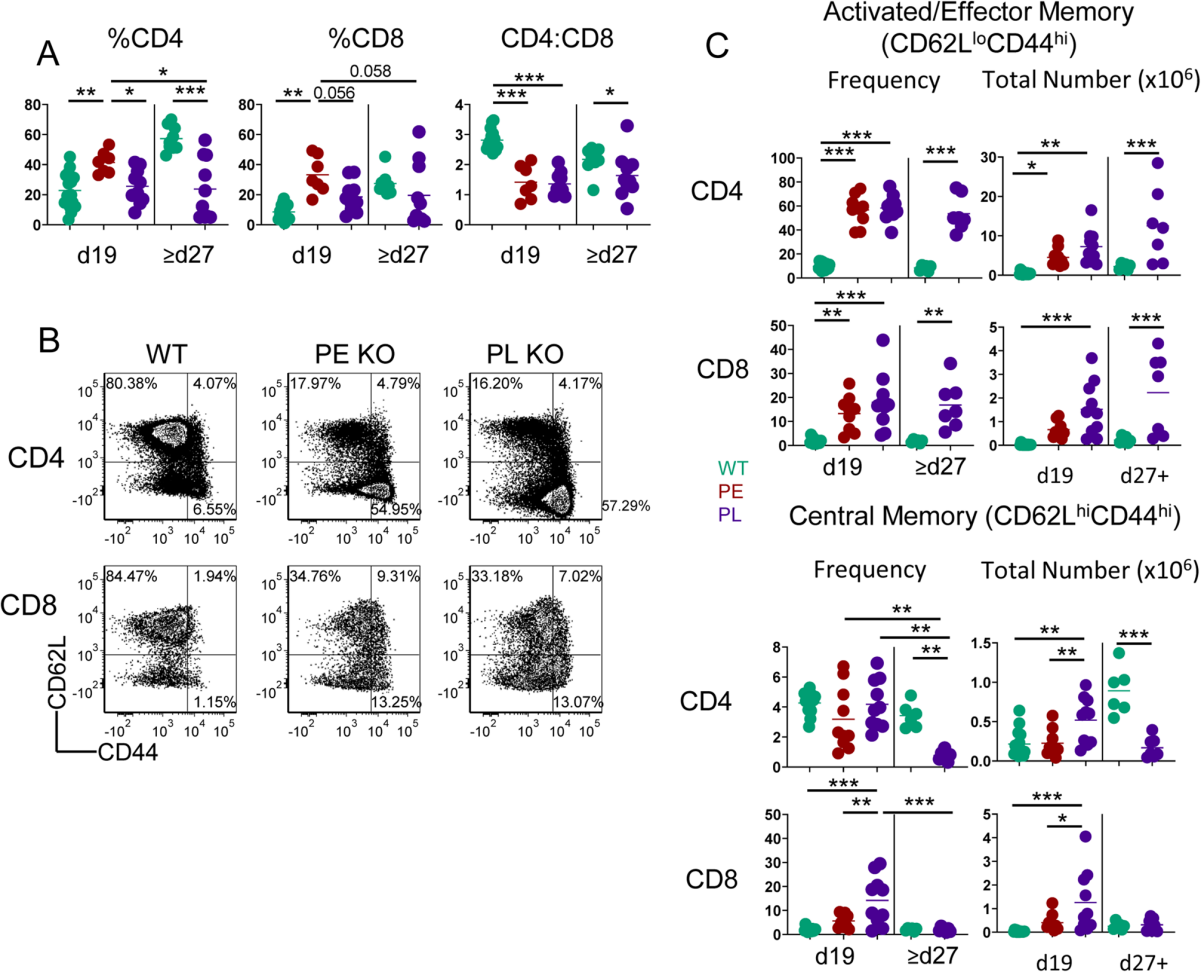
IL-2Rα-KO T cells express markers indicative of developing memory. (**A**) CD4 and CD8 T cell frequency of live B220-CD11b-CD11c-Gr-1-cells and the ratio of CD4:CD8 T cells in spleen are shown for 19-day-old and late endpoint (d27+) WT and IL-2Rα-KO mice. (**B**) Representative flow plots of CD44 and CD62L expression on spleen CD4 and CD8 T cells in 19-day-old WT and IL-2Rα-KO mice. (**C**) Frequency and total number of CD4 and CD8 T cell activation state in 19-day-old and late endpoint (d27+) WT and IL-2Rα-KO mice. Statistics: one-way ANOVA with Benjamini–Hochberg FDR correction; *p < 0.05, **p < 0.01, ***p < 0.001; *n* = 5–10 mice per experimental group. Non-significant comparisons are not shown. For total number, comparisons between mice of different ages are not shown to due differences in mouse size.

Lymphoproliferation and T cell activation occur in IL-2-KO and IL-2Rα-KO mice^9,10^, however the preceding events and immune parameters that correlate with early disease onset are unknown. To identify such immune phenotypes, mice were evaluated early in disease onset (day 19) and separated into predicted PE or PL disease groups based on four-parameter PCA and assessed for T cell numbers and activation by flow cytometry. CD4:CD8 ratio in the spleen and BM of both disease groups shifted from an expected ratio of ~ 2:1 to 1:1, although PL mice had a lower frequency of splenic CD4 and CD8 T cells than PE mice, which was not associated with a similar change in total number (Fig. 2A, Supplementary Figs. S2A, S3A). The shift in CD4:CD8 T cell ratio is not a consequence of thymic development, as thymic T cell ratios were normal (Supplementary Fig. S2B).

PE and PL IL-2Rα-KO disease groups demonstrated increased T cell activation based on reduced CD62L and elevated CD44 expression, as previously observed in IL-2-KO mice (Fig. 2B,C, Supplementary Fig. S2C). CD8 T cells exhibited markers indicative of an expanded developing central memory population (CD62L^hi^CD44^hi^), and expansion of this CD8 T cell memory population in the spleen associated with late disease kinetics (Fig. 2B,C). Strikingly, splenic CD4 and CD8 T cell central memory was significantly decreased in mice at late disease endpoint (Fig. 2C). These results indicate that a fairly normal splenic T cell frequency and memory T expansion are associated with slower disease development. Conversely, increased T cell splenic frequencies with normal frequencies of memory T cell populations are associated with rapid disease progression.

### IL-2Rα-KO mice exhibit kinetic-dependent differences in CLP frequency

**Figure 3:**
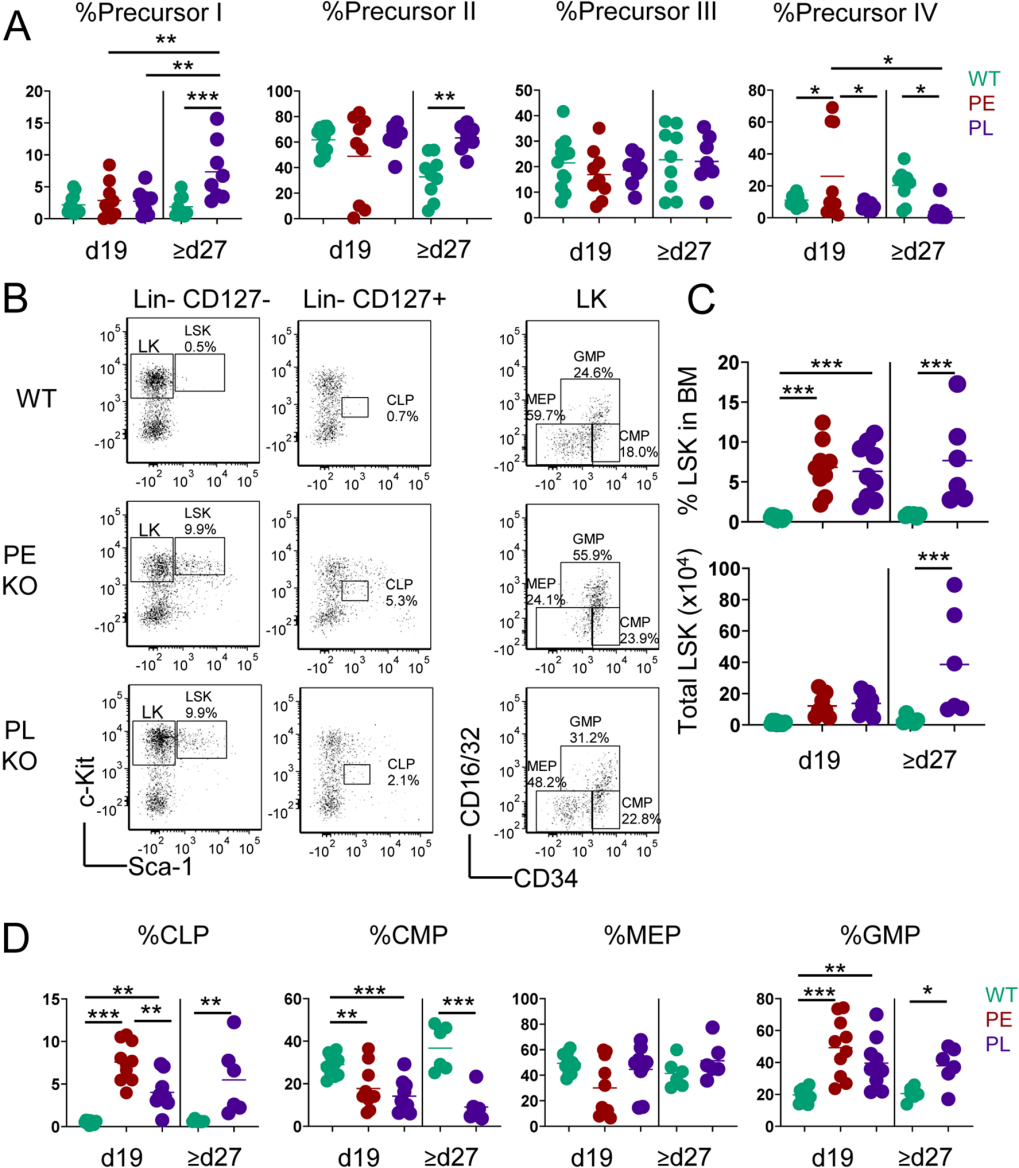
IL-2Rα-KO mice exhibit kinetic-dependent differences in CLP and RBC precursor frequency. (**A**) Frequency of RBC precursor analysis by expression of CD71 and Ter119 in WT and IL-2Rα-KO mice at day 19 and day ≥ 27. (**B**) Representative flow plots for hematopoietic progenitor populations in BM of 19-day-old WT and IL-2Rα-KO mice. (**C**) Frequency and total number of LSK cells in 19-day-old and late endpoint (d27+) WT and IL-2Rα-KO mice. (**D**) Frequency of hematopoietic progenitor populations in 19-day-old and late endpoint (d27+) WT and IL-2Rα-KO mice. Statistics: one-way ANOVA with Benjamini–Hochberg FDR correction; *p < 0.05, **p < 0.01, ***p < 0.001; *n* = 9–12 mice per experimental group. Non-significant comparisons are not shown. For total number, comparisons between mice of different ages are not shown to due differences in mouse size.

Mice lacking IL-2 also develop severe BMF contributing to anemia development^11,23^. We evaluated whether there were differences in BM stem cell and progenitor populations between PE and PL IL-2Rα-KO mice. Since IL-2Rα-KO mice die from hypoxia, dysregulated RBC development is critical to disease endpoint^11^. As such, we assessed RBC precursor populations, which gain Ter119 and lose CD71 expression as they mature, moving from precursor I (Ter119^int^CD71^high^) progressively to the precursor IV stage (Ter119^hi^CD71^neg^)^24^. At day 19, no deficiencies in RBC development and maturation were observed, although there is an increase in precursor IV cells in some PE mice (Fig. 3A, Supplementary Fig. S3B), that does not translate to changes in the megakaryocyte-erythrocyte progenitors (MEP; Fig. 3D). In contrast to IL-2-KO disease, we observe no major alteration in IL-2Rα-KO BM erythrocyte precursors during early endpoint disease, although some changes were observed in late stage endpoints, despite similar physical signs of anemia, which includes an increase in precursors I/II, but a decrease in later-stage RBC progenitors (precursor IV) (Fig. 3A, Supplementary Fig. S3B). These data suggest that early disease is not driven by RBC progenitor defects but may be important in late end-stage disease.

Next, PE and PL IL-2Rα-KO and WT BM was evaluated for hematopoietic progenitors (Fig. 3B,C). IL-2Rα-KO mice in both groups exhibited increased LSK frequency and total number and decreased common myeloid progenitors (CMP) compared to WT (Fig. 3C,D, Supplementary Fig. S3C). Common lymphoid progenitors (CLP) and granulocyte monocyte progenitor (GMP) frequency, but not total number, was increased compared to WT (Fig. 3D, Supplementary Fig. S3C). In IL-2-KO mice, expanded LSKs were less competitive relative to WT LSKs, driving a BMF phenotype^11^. Similar to IL-2-KO mice, BM progenitor frequency is altered in IL-2Rα-KO mice. Importantly, CLP frequency was higher in PE versus PL mice, perhaps contributing to increased splenic CD4 and CD8 T cell frequency in these mice.

### Unchanged thymic development in IL-2Rα-KO mice

To determine whether changes in BM CLP frequency translated to changes in thymic T cell development, we assessed thymic T cell populations and thymic CLP frequency in WT and IL-2Rα-KO mice. Despite increases in BM CLP, thymic CLP frequencies and numbers were unchanged at both day 19 and late endpoint (Supplementary Fig. S4A). In agreement with previous work on IL-2Rα-KO mice^10^, there were no significant changes to developing T cell populations, including single-positive, double-positive, and double-negative (Supplementary Fig. S4B,C). This data suggests that all changes to peripheral T cell population frequencies are due to changes in the periphery, not from changes during development.

### IL-2Rα-KO Tregs acquire effector functionality

**Figure 4:**
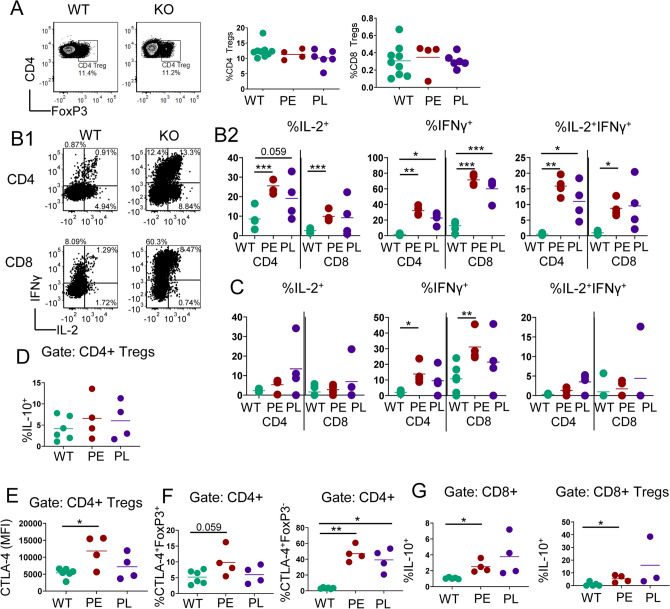
IL-2Rα-KO Tregs acquire effector functionality. (**A**) Representative flow plots, and frequency of CD4 and CD8 Tregs in the spleen in 19-day-old WT and IL-2Rα-KO mice. *n* = 4–11 mice per experimental group. Representative flow plots and frequency of T cells producing IL-2 and IFNγ in 19-day-old WT and IL-2Rα-KO LN T cells (**B1**,**B2**) or Tregs (**C**) post 5-h PMA and ionomycin stimulation. *n* = 4–6 mice per experimental group. (**D**) CTLA-4 expression on CD4 Treg cells at day 19 by mean fluorescent intensity (MFI). (**E**) Frequency of IL-10+ 19-day-old WT and IL-2Rα-KO CD4 Treg cells post 5-h PMA and ionomycin stimulation. (**F**) Frequency of CTLA-4+FoxP3+CD4 T cells or CTLA-4+FoxP3− CD4 T cells are shown for 19-day-old WT and IL-2Rα-KO mice. (**G**) Frequency of IL-10+ 19-day-old WT and IL-2Rα-KO CD8 T and CD8 Treg cells post 5-h PMA and ionomycin stimulation. *n* = 4–6 mice per experimental group over 4 independent experiments. Statistics: unpaired Student’s t test; *p < 0.05, **p < 0.01, ***p < 0.001. Non-significant comparisons are not shown.

A major defect in IL-2-KO and IL-2 signaling deficient mice is a reduction in CD4 Treg frequency and function, so we assessed whether disease outcome was associated with Treg frequency^3,25–27^. Surprisingly, splenic IL-2Rα-KO CD4 and CD8 Treg frequency was comparable to WT (Fig. 4A, Supplementary Fig. S5A)^3^.

We next evaluated whether disease kinetics was determined by cytokine production or differential signaling in response to cytokines. Elevated serum IL-2 levels have been observed in C57BL/6 IL-2Rα-KO mice^28^, and IFNγ is critical for early autoimmune disease in IL-2-KO mice^11,12,23,29,30^. A significant frequency of IL-2Rα-KO CD4 and CD8 T cells produced IL-2, IFNγ, and a combination of both cytokines, however this frequency was similar between PE and PL IL-2Rα-KO mice (Fig. 4B1,B2). A higher frequency of PE IL-2Rα-KO CD4 and CD8 Treg cells also expressed IFNγ compared to WT, suggesting a gain of effector function (Fig. 4C, Supplementary Fig. S5B,C). FoxP3+ Tregs conversion into effector cells has been demonstrated under inflammatory conditions, including autoimmune disease^31–33^. To determine whether IL-2Rα-KO CD4 Treg cells retain a suppressive phenotype, we assessed IL-10 production and CTLA-4 expression. WT and IL-2Rα-KO mice had similar IL-10+ CD4 Treg frequencies (Fig. 4D), but CTLA-4 expression was slightly elevated in PE compared to WT CD4 Treg cells (Fig. 4E). Together this data suggests that IL-2Rα-KO CD4 Treg cells have acquired effector capacity while largely retaining CTLA-4 expression. Strikingly, CTLA-4 was primarily expressed on FoxP3− CD4 T cells in IL-2Rα-KO (Fig. 4E). Additionally, we found an increase in IL-10 producing CD8 T and CD8 Treg cells in PE mice and PL mice compared to WT (Fig. 4G), although PL increases were not significant due to the larger spread of the data.

### Elevated IL-2Rβ expression on IL-2Rα-KO CD8 T cells

**Figure 5:**
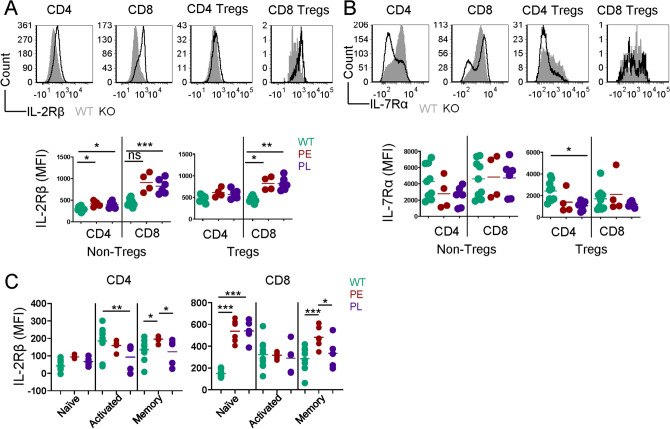
IL-2Rα-KO CD8 T cells express two-fold higher levels of IL-2Rβ than WT CD8 T cells. Representative histograms showing the expression and summary of mean fluorescent intensity (MFI) of (**A**) IL-2Rβ and (**B**) IL-7Rα on CD4, CD8, CD4 Treg, and CD8 Treg cells in spleen of 19-day-old WT and IL-2Rα-KO mice. *n* = 5–10 mice per experimental group. (**C**) MFI of IL-2Rβ on LN cells from 19-day-old WT and IL-2Rα-KO mice based on activation state as defined in Fig. 2B. *n* = 5–10 mice per experimental group. Statistics: unpaired Student’s t test; *p < 0.05, **p < 0.01, ***p < 0.001. Non-significant comparisons are not shown.

Cytokines IL-2, IL-7, and IL-15 provide necessary survival signals to T cells^34,35^. Elevated IL-7Rα expression on IL-2-KO CD4 T cells promotes autoimmunity^18,36,37^. To assess whether disease outcome was associated with differences in T cell capacity to respond to these cytokines, IL-2Rβ and IL-7Rα expression on T cells was evaluated. IL-2Rβ expression was elevated on IL-2Rα-KO CD4, CD8, and CD8 Treg cells in the spleen compared to WT (Fig. 5A). Differences in PE and PL CD8 T cell expression of IL-2Rβ were clearly observed in naïve and central memory T cells, but not in activated/effector memory cells (Fig. 5C). IL-7Rα expression was mildly decreased on splenic CD4 Tregs from PL mice (Fig. 5B). Differences in IL-2Rβ expression observed in CD4 and CD8 T cells in IL-2Rα-KO mice were not explained by disease kinetics. Cytokine receptor expression levels indicate a differing capacity for CD4 and CD8 T cell responses to IL-2 and IL-15 in the absence of IL-2Rα signaling, suggesting a possible mechanism for disease kinetics.

### IL-2Rα-KO T cells are differentially capable of responding to IL-2

**Figure 6:**
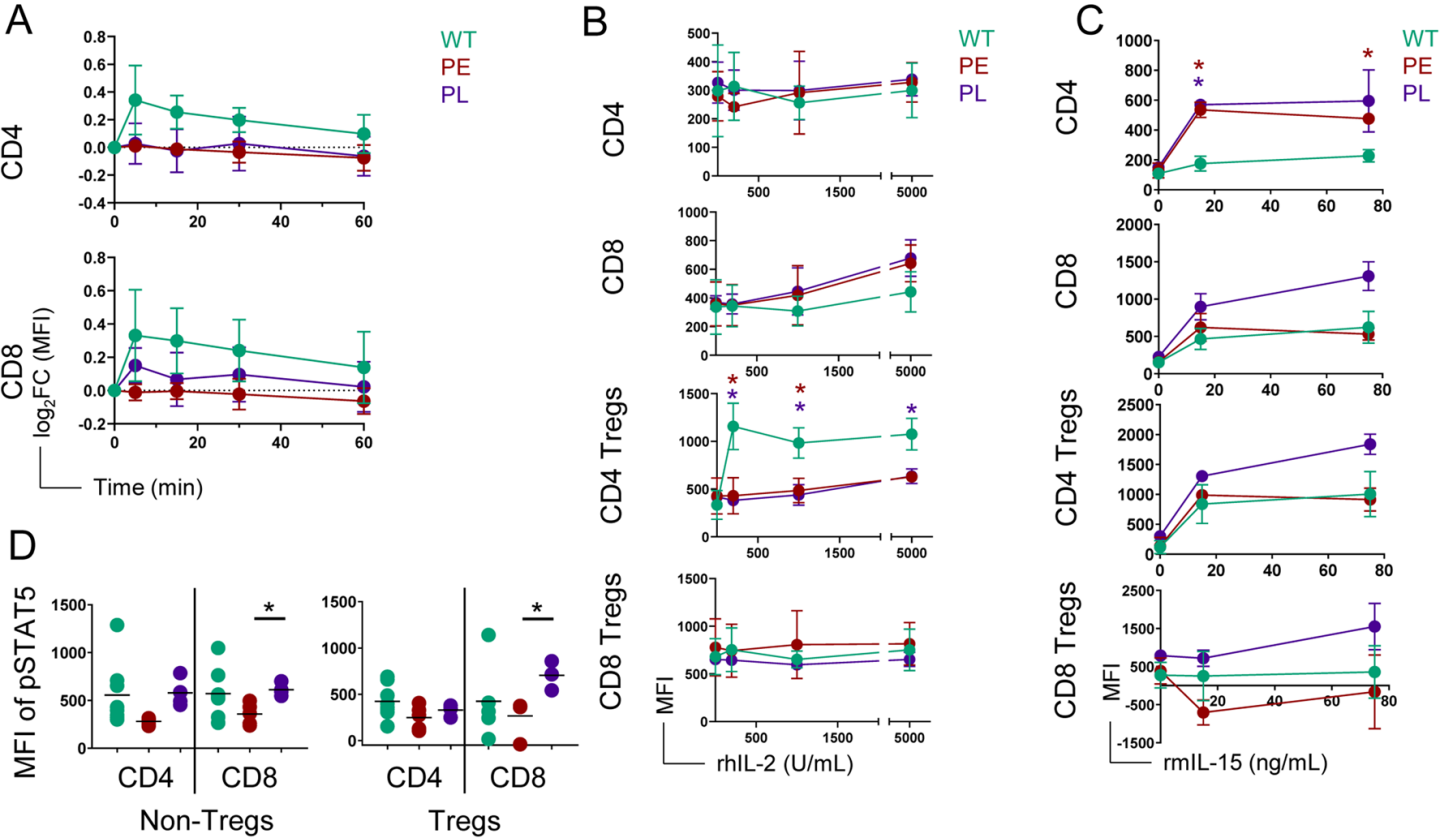
IL-2Rα-KO T cells respond differentially to cytokine stimulation. (**A**) Log_2_ fold change of S6 phosphorylation by mean fluorescent intensity (MFI) from unstimulated over time following TCR stimulation comparing WT versus IL-2Rα-KO mice for CD4 and CD8 T cells. *n* = 2–4 mice per experimental group from 5 independent experiments. (**B**) MFI of phosphorylated STAT5 in 19-day-old WT and IL-2Rα-KO splenic T cells stimulated with rhIL-2 for 15 min with the indicated dosage. *n* = 2–3 mice per experimental group. (**C**) MFI of phosphorylated STAT5 in 19-day-old WT and IL-2Rα-KO splenic T cells stimulated with rmIL-15 for 15 min with the indicated dosage. *n* = 2–4 mice per experimental group from 2 independent experiments. (**D**) MFI of STAT5 phosphorylation in 19-day-old WT and IL-2Rα-KO T cells following 10 ng/mL rmIL-7 stimulation for 15 min. *n* = 4–8 mice per experimental group. Statistics: unpaired Student’s t test; *p < 0.05, **p < 0.01, ***p < 0.001. Non-significant comparisons are not shown. Statistics in (**B**) and (**C**) are compared to WT and colored by which IL-2Rα-KO group is being compared.

IL-2 signaling in T cells lowers the threshold of TCR stimulation needed for CD8 T cell proliferation, and higher CD8 T cell basal IL-2Rβ expression allows for sustained IL-2 signaling, leading CD8 T cells to proliferate earlier than CD4 T cells^16,38^. Since IL-2Rα-KO CD8 T cells have two-fold higher IL-2Rβ expression than WT CD8 T cells (Fig. 5A), we evaluated the signaling capacity of IL-2Rα-KO T cells. To assess whether the lack of IL-2Rα would impact TCR signaling, we measured ribosomal protein S6 phosphorylation following TCR stimulation in splenocytes. We found no significant differences in response to TCR stimulation (Fig. 6A). IL-2Rα-KO T cell response to IL-2 was assessed by STAT5 phosphorylation. High IL-2 levels utilized for these stimulations exist in IL-2Rα-KO mice^28^. IL-2Rα-KO CD4 Treg cells were less capable of signaling in response to IL-2 regardless of disease kinetics (Fig. 6B). However, STAT5 phosphorylation in response to IL-2 was similar between WT and IL-2Rα-KO CD8 T cells at all doses, perhaps because elevated IL-2Rβ compensates for absence of IL-2Rα on IL-2Rα-KO cells (Fig. 6B). WT and IL-2Rα-KO CD4 T and CD8 Treg cells were non-responsive to IL-2, as previously observed for CD4 T cells in ex vivo re-stimulations^16^. Since IL-15 shares receptor subunits with IL-2, we assessed whether IL-2Rα-KO T cells respond differentially to IL-15 via STAT5. CD8 T, and CD4 and CD8 Treg cells responded similarly to IL-15 stimulation between WT and IL-2Rα-KO mice, while IL-2Rα-KO CD4 T cells were more responsive to IL-15 stimulation than their WT counterparts (Fig. 6C). PL IL-2Rα-KO CD8 T, CD4 Treg, and CD8 Treg cells were also more responsive than PE counterparts (Fig. 6C), although these differences were smaller and non-significant.

Splenic IL-2Rα-KO CD4 Treg cells had increased IL-7Rα surface expression compared to WT (Fig. 5B). To determine whether this receptor difference resulted in a differential response to IL-7, we assessed pSTAT5 levels. Surprisingly, given similar IL-7Rα expression, PE IL-2Rα-KO CD8 T and CD8 Treg cells exhibited reduced IL-7 responsiveness (Fig. 6D). Despite differences in IL-7Rα expression on splenic CD4 Treg cells, responsiveness to IL-7 was unchanged (Fig. 6D).

**Figure 7:**
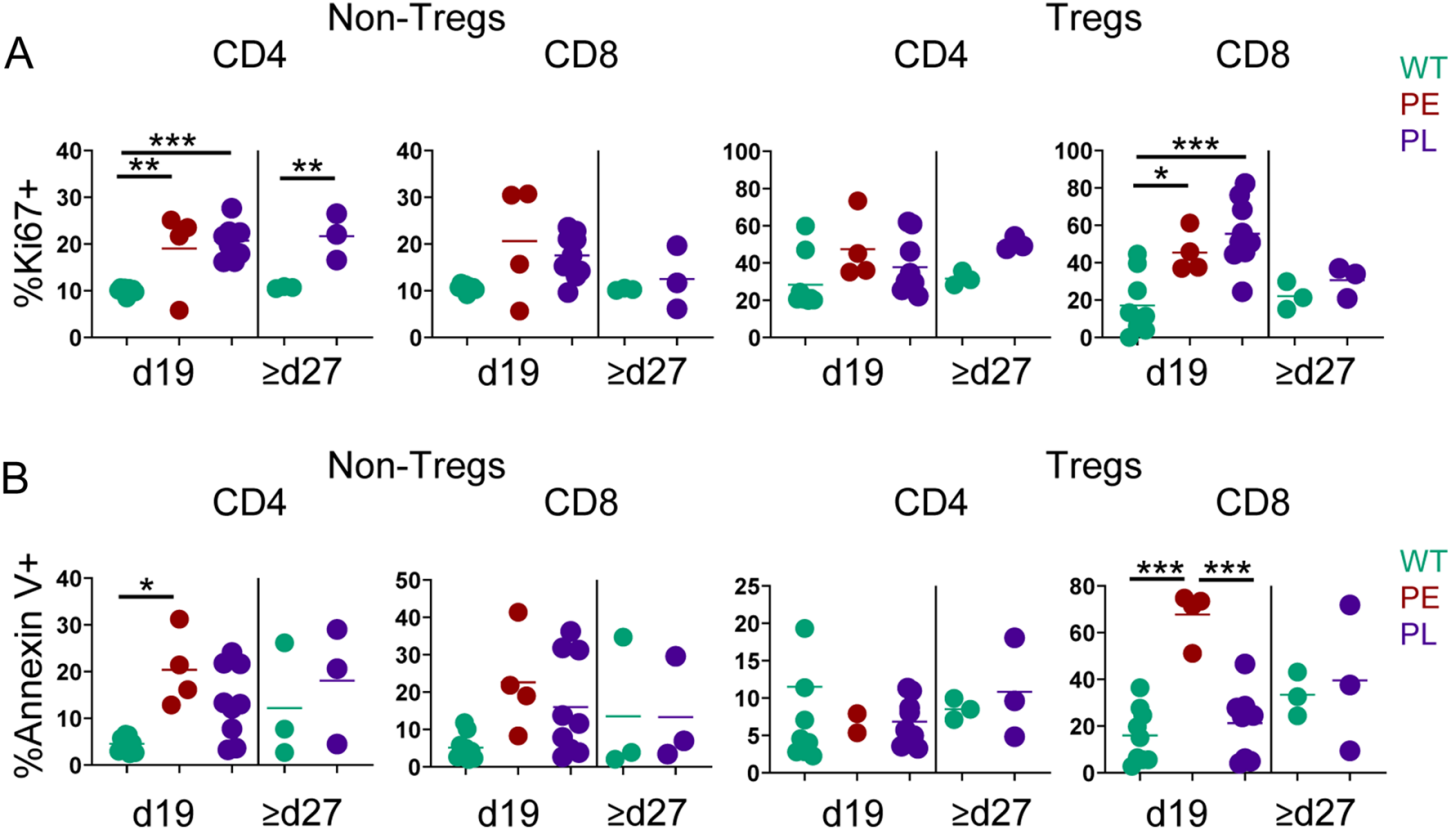
IL-2Rα-KO T cells exhibit differential proliferation and apoptosis. Frequency of (**A**) Ki67+ or (**B**) annexin V+ 19-day-old and late endpoint (d27+) WT and IL-2Rα-KO T cells from the spleen are shown. *n* = 3–9 mice per experimental group. Statistics: one-way ANOVA with Benjamini–Hochberg FDR correction; *p < 0.05, **p < 0.01, ***p < 0.001. Non-significant comparisons are not shown.

To evaluate whether differential signaling outcomes impact T cell proliferation and apoptosis, Ki67 and Annexin V expression was assessed directly ex vivo. IL-2Rα-KO CD4 T and CD8 Treg cells proliferated more than WT T cells (Fig. 7A,B). Further, PE IL-2Rα-KO CD4 T and CD8 Treg cells exhibited enhanced apoptosis (Fig. 7A,B). The increased apoptosis seen in PE IL-2Rα-KO CD4 T cells may account for the greater frequency of PE IL-2Rα-KO CD8 T cells. Increased apoptosis of PE IL-2Rα-KO CD8 Treg cells may contribute to more rapid disease kinetics.

While the primary site of hemolytic anemia is the spleen, we also performed staining and functional assessments on lymphocytes as disease in IL-2Rα-KO and IL-2-KO mice is systemic and lymph nodes are a site of T cell activation. While much of what we found in the lymph nodes mirrored that seen in the spleen, there were distinct changes that were present in the lymph nodes that were not present in the spleen. A higher frequency of IL-2Rα-KO lymph node CD4 T cells from PL mice exhibited an emerging memory population (CD62L^hi^CD44^hi^) relative to PE mice, as is seen with CD8 T cells in the spleen (Fig. 2C, Supplementary Fig. S6A). As is expected in this model, CD4 and CD8 Tregs are reduced in frequency in the lymph node, contrasting what is seen in the spleen (Fig. 4A, Supplementary Fig. S6B)^3^. IL-10-producing CD8 T and CD8 Treg cells were reduced in PE IL-2Rα-KO mice compared to PL and WT, suggesting reduced suppression at these sites of T cell activation (Supplementary Fig. S6C). While TCR responses were unchanged in the spleen, PE lymphocytes had less sustained and weaker peak phosphorylation and PL lymphocytes had slower kinetics and drastically reduced maximum phosphorylation (Supplementary Fig. S6D).

**Figure 8:**
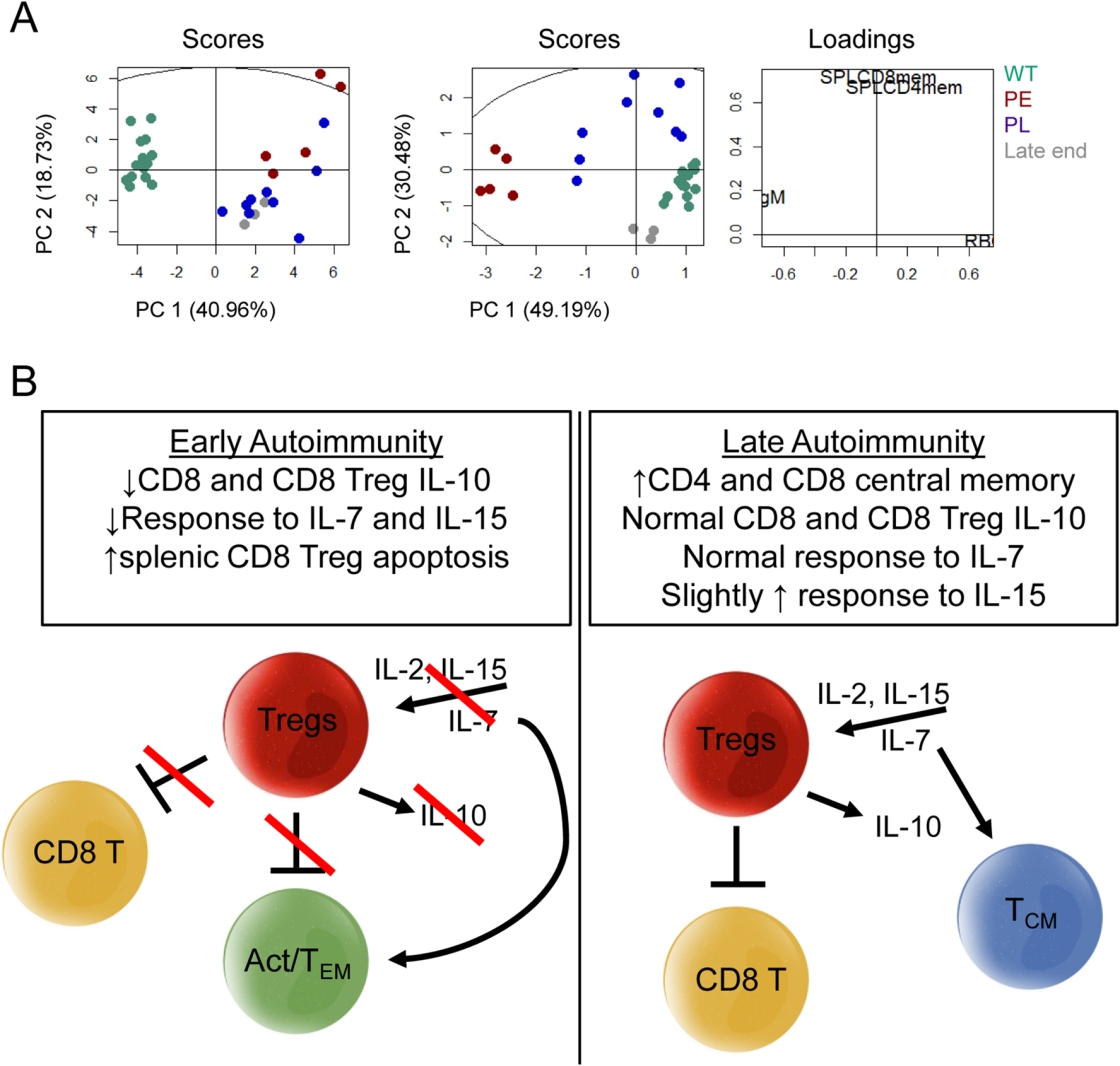
PE and PL IL-2Rα-KO disease are distinct. (**A**) PCA was performed on 35 variables (left) collected from the same mice including RBC, hemoglobin, hematocrit, RBC-bound IgM and IgG, and data shown in Figs. 2, 3 and Supplementary Fig. S1. IL-2Rα-KO mice at day 19 and late endpoint, along with correspondingly aged WT controls were used for PCA. Following regression tree analysis PCA was performed on five variables: RBC, RBC-bound IgM, splenic CD4 central memory T cells, and CD8 central memory T cells (right). Percentage shown on axes refers to the amount of variance in the data that is explained by the given principal component. (**B**) Summary of PE vs PL disease progression; all changes are relative to WT activities. In PE IL-2Rα-KO mice, lowered T cell responsiveness to IL-2, IL-7, and IL-15 leads to reduced function of Tregs (reduced IL-10 production) and increased apoptosis of CD8 Tregs, allowing T cell effector activation (act/T_EM_). In PL IL-2Rα-KO mice, normal to slightly elevated IL-2, IL-7, and IL-15 responsiveness allows for prolonged Treg function. Enhanced responsiveness to IL-15 and IL-2 may be driving central memory (T_CM_) differentiation in mice with delayed disease.

Finally, to assess how different PE and PL disease are from each other, PCA was performed on 35 parameters gathered from the same mice, including data from the spleen, bone marrow, peripheral blood, and lymph nodes. WT and IL-2Rα-KO mice clearly separate, as expected; however, PE and PL IL-2Rα-KO mice did not (Fig. 8A). Following regression tree analysis to identify the most useful separating variables, PCA was performed again. As before, WT and IL-2Rα-KO mice were separated, but importantly PE and PL IL-2Rα-KO mice were also distinct (Fig. 8A). In the refined PCA analysis PE, PL, and late endpoint IL-2Rα-KO mice separated from each other. Interestingly, PL on day 19 and PL endpoint (end stage disease) overlap almost completely in PC1, but separate in PC2, suggesting that some factors (PC1) drive disease induction while other factors (PC2) change during late stages of autoimmunity. This demonstrates that the disease in early and late kinetics have many overlapping features, but when most significant variables are assessed, PE and PL disease are distinct even through to disease endpoint when signs of anemia are similar (Fig. 1H).

## Discussion

Several perturbations combine to delay or accelerate disease in the absence of appropriate IL-2 signals. This study identified several small alterations that together may contribute to differences in disease kinetics. IL-2Rα-KO mice with delayed disease kinetics (PL) had increased central memory T cells and some maintained CD8 Treg IL-10 expression. In contrast, PE IL-2Rα-KO T cells displayed reduced IL-7 signaling. Increased CLP output in PE mice along with elevated proliferation, normal TCR signals, and reduced suppression from CD8 Tregs may provide an environment that more rapidly induces disease onset and progression. As the spleen is the primary site of AIHA, higher memory T cell frequency, expanded Treg effector responses, and enhanced proliferation may further contribute to accelerated death in PE IL-2Rα-KO mice. Importantly end stage disease is not identical for early and late death. While anemia pathologies were similar, several differences remained in the underlying mechanisms driving disease kinetics and endpoint pathologies (Fig. 8A).

Follicular CD4 and CD8 T cells promote antibody production in autoimmune disease^39,40^. In IL-2-KO mice, CD8 T follicular cells increase dramatically shortly before disease endpoint^19^. Like CD4 T helper cells, CD8 T follicular cells promote plasma cell differentiation, antibody production and class-switching^19,41–43^. CD8 T follicular cells were mildly elevated but non-significant in PE compared to PL IL-2Ra-KO mice. This CD8 T follicular cell increase near disease endpoint in IL-2Rα-KO mice may accelerate endpoint disease progression by contributing to elevated anti-RBC antibody production. Differentiation signals for CD8 T follicular cells are currently unknown, however, these cells express high IL-7Rα^44^, and thus their expansion in PE mice may be due to altered IL-2 and IL-7 signaling.

In addition to AIHA, IL-2-KO mice develop severe BMF that contributes to severe anemia^11,23^. We observed defects in IL-2Rα-KO BM indicative of BMF, including increased frequencies of LSK, CLP, and GMP, and reduced CMPs. Surprisingly these alterations did not translate into early RBC precursor defects in IL-2Rα-KO mice, regardless of disease kinetics. RBC precursor defects only became apparent in older IL-2Rα-KO mice, suggesting that BMF only mildly contributes to early anemia but may be relevant to late disease kinetics, despite BMF contribution to rapid disease in IL-2-KO mice. This delayed contribution of BMF to overall anemia in PL mice is likely due to normal MEP frequency during early stages of IL-2Rα-KO disease. The reversed CD4:CD8 ratio in IL-2Ra-KO mice could be due to increases in CLPs. However, thymic CD4:CD8 T cell ratio is normal indicating that this shift is likely due to peripheral influences. CD8 T cells with higher IL-2Rβ might out-compete CD4 T cells for cytokine signals, thus surviving more and skewing the CD4:CD8 T cell ratio.

Activated and developing central memory CD4 and CD8 T cells were found in PL disease similar to C57BL/6 mice that develop autoimmunity with delayed kinetics^28,45–47^. However, a corresponding increase in the memory marker, IL-7Rα, on CD4 or CD8 T cells was not consistently seen, contrasting IL-2-KO mice, perhaps due to difference in signaling when IL-2 is absent rather than highly expressed^18,45^. Although elevated IL-2Rβ expression did not correspond to disease kinetics, this pattern is indicative of memory CD8 T cells, which were increased in IL-2Rα-KO mice. IL-2Rα-KO Tregs receive reduced IL-2 signals, in spite of elevated IL-2 availability, lymph node Tregs, although Treg frequency is normal in the spleen^28^. Increased IL-2Rβ expression on CD8 T cells may provide an edge on responding to IL-2 over CD4 T cells. However, normal IL-2 signaling in CD8 T cells along with elevated IL-2 production would indicate an environment that inhibits differentiation of CD8 T cells into central memory, and favors effector and effector memory differentiation^7,28^. Yet, our results and previous studies phenotypically indicate that IL-2Rα-KO CD8 T cells are developing into central memory-like cells^28^. This apparent contradiction could be due to the timeframe in which we assessed IL-2 signaling. CD8 T cells can maintain IL-2 signaling over several hours, a trait that CD4 T cells do not share^38^. It is possible that if assessed at later timepoints of IL-2 signaling, IL-2Rα-KO CD8 T cells may be defective and permissive for memory differentiation, or constant exposure to elevated IL-2 in vivo may skew T cell differentiation or signaling feedback. Increased frequency of central memory-like T cells in PL IL-2Rα-KO mice may contribute to delayed disease progression, as these cells are thought to have a lower effector function than other activated T cells^45,47^.

Elevated serum IL-2 likely contributes to an increased memory CD8 T cell population in IL-2Rα-KO mice^28^. Supporting this, a higher frequency of IL-2Rα-KO T cells produce IL-2 than WT T cells and mice maintain normal splenic CD4 and CD8 Treg frequencies. IL-2Rα-KO CD4 Tregs expressed normal IL-2Rβ and were less responsive to IL-2 but maintained normal responsiveness to IL-15. Tosiek et al. suggested that CD4 Treg responsiveness to IL-15 allows survival in tissues with lower IL-2 accessibility^48^, such as the spleen^49^, possibly contributing to increased IL-2Rα-KO splenic Treg proliferation and frequency. Alternatively, maintenance of normal Treg frequency may be due to expansion of Tregs that acquired effector function, including IFNγ production. Disease in IL-2Rα-KO and IL-2-KO mice is IFNγ-mediated^11,12,30^, thus IL-2Rα-KO Tregs may be actively contributing to disease pathology.

IL-2 is required for the maintenance and survival of CD4 Tregs^50,51^ and IL-2 or receptor loss leads to reduced Tregs and autoimmunity onset^3,8–10,52^. CD4 Treg transfer before autoimmunity onset prevents autoimmune development, including autoimmunity within IL-Rβ-KO mice^27,53^, which speaks to the importance of CD4 Tregs in preventing autoimmunity. However, these studies did not test CD8 Treg transfer or assess how CD4 Tregs affect autoimmune disease progression after onset. CD8 Tregs function in suppressing CD8 T cell responses, memory T cell responses, autoantibody production from CD4 T follicular helper cells, and multiple organ immune infiltration causing autoimmunity^54–58^. We observed that PL IL-2Rα-KO CD8 Tregs respond better to IL-7 and produce more IL-10 than their PE counterparts, perhaps allowing for maintenance of suppressive control. Additionally, in EAE and collagen-induced arthritis models, transfer of regulatory B cells, CD4 Tregs, or CD8 Tregs, prevents autoimmunity^59–64^. In EAE, the in vivo role of regulatory B cells and CD4 Tregs differs by the stage of disease progression. These suggest that defects in suppression from multiple regulatory cells may lead to more severe autoimmunity as layers of control on T and B cell activation are lost. Gain of IFNγ production and reduction in IL-10 production by CD8 Tregs in PE IL-2Rα-KO mice may allow earlier advancement of autoimmunity.

Rapid disease in IL-2Rα-KO mice is likely driven by disruption of T effector and Treg balance. PE and PL IL-2Rα-KO CD4 and CD8 Tregs acquire effector cytokine expression. PE CD8 Tregs have increased apoptosis, and reduced IL-10 expression in lymph node CD8 T cells and Tregs, resulting in reduced Treg suppressive capacity at the site of T cell activation. As there are several CD8 Treg subsets (some not defined by Foxp3 expression)^65,66^, IL-10 production by CD8+ Foxp3− could represent expression by other subsets, or by conventional CD8 T cells. This data suggests that CD8 T cell IL-10 production may contribute to disease kinetics although CD4 Tregs are critical for overall autoimmune development. As effector T cells and Tregs compete for survival signals, changes in receptor abundance and signaling strength, and local cytokine encounters, may tip the balance between Treg suppression and effector expansion, leading to rapid, early disease in PE IL-2Rα-KO mice.

IL-2 reduces the threshold of TCR signaling needed to induce proliferation in CD8 T cells, but not CD4 T cells^16^. Increased IL-2Rβ expression on CD8 T cells leads to sustained IL-2 signaling and earlier proliferation induction^38^. IL-2Rα-KO lymph node CD4 and CD8 T cells were significantly less responsive to TCR signaling than WT CD4 and CD8 T cells respectively, but with differing kinetics between PE and PL mice, perhaps suggesting that PL T cells maintain a less responsive state delaying disease. STAT5 phosphorylation in IL-2Rα-KO T cells following IL-2 stimulation indicated that while CD4 Tregs were less responsive to lower IL-2 than their WT counterparts, IL-2Rα-KO CD4 and CD8 T cells were not impaired in their ability to respond to IL-2. IL-2Rα-KO CD8 T cells express higher IL-2Rβ than WT CD8 T cells, perhaps allowing higher IL-2 sensitivity and responsiveness. Indicative of an effector CD8 phenotype that promotes rapid disease kinetics, PL CD4 and CD8 T cells responded normally following IL-7 stimulation, while PE T cells were less responsive. Altered cytokine signaling, from IL-2Rβ upregulation and increased IL-2 production, along with increased apoptosis and reduced IL-10 production in CD8 Tregs, may in turn promote elevated CD8 T cells and effector/memory fate choices that skew disease kinetics (Fig. 8B). Meanwhile in WT mice, self-reactive T cells would be suppressed by normal Treg function, preventing massive proliferation and autoimmune manifestation.

## Materials and methods

### Mice

All animal procedures and protocols were approved by the UC Merced Animal Care and Use Committee (protocol number AUP18-0005). Animal euthanasia followed the Guide for the Care and Use of Laboratory, and humane endpoints were used in survival studies. BALB/c IL-2Rα-KO or control littermate mice (wildtype and heterozygous; WT) were bred and maintained in our specific pathogen-free facility in an Animal Barrier Facility in accordance with the guidelines of the Laboratory Animal Resource Center of the University of California Merced and under approval by the Institutional Animal Care and Use Committee with appropriate training for all animal husbandry and experimentation. Disease manifestation and survival of IL-2Rα-KO and littermate controls was monitored daily from day 9 onward, with a subset assigned body scores based on signs of anemia (Supplementary Table S1), with 80 days being the longest an animal was monitored before endpoint reached. Signs of severe autoimmune disease include hunched appearance, lethargy, ruffled hair, and hypoxia, and are indicative of endpoint within 4–8 h. Mice displaying these signs of autoimmune disease manifestation were euthanized and age of death presumed to be within the day.

### Complete blood count (CBC)

Blood samples used for predictions were collected from mice aged 19 days via the submandibular vein. Endpoint blood collection was performed via terminal eye bleeds immediately following carbon dioxide asphyxiation into heparinized tubes^52^. CBC were evaluated within 8 h on a Hemavet 950 Veterinary Hematology System (Erba Diagnostics). Standard CBC medical abbreviations are used in all figures and text^67^.

### RBC Ab detection

Abs bound to RBCs were detected using flow cytometry as previously described^18^. RBCs were freshly isolated from mice by terminal bleed or submandibular vein puncture, washed three times in room-temperature PBS, and resuspended to 1% RBCs. 10 μl of 1% RBCs were incubated with either anti-mouse IgM-FITC (1:100 dilution; on ice; Jackson ImmunoResearch) or anti-mouse IgG-FITC (1:50 dilution; at 37 °C; Jackson ImmunoResearch) for 20–30 min. The percentage of RBCs bound by Ab was determined by flow cytometry.

### Flow cytometry

Abs were purchased from eBioscience and staining was performed in PBS/1%FBS (Omega Scientific) for 30 min at 4 °C unless otherwise noted. Cell viability was determined using eFluor506 viability dye (eBioscience). To evaluate cytokine receptor expression on T cells, both Treg and non-Treg, lymph node and splenic cells were incubated with surface antibodies prior to fixation. Cells were fixed using the FoxP3/Transcription Factor Staining Buffer Kit (Invitrogen) according to manufacturer’s instructions. Following fixation cells were stained intracellularly for FoxP3 (FJK-16s). To evaluate hematopoietic progenitor populations in the BM and thymus, RBC-lysed BM and thymic cells were first incubated with biotinylated lineage/dump markers without Fc-block, washed, then stained for surface markers and streptavidin. To evaluate RBC precursors, whole BM cells were incubated with anti-Ter119 (TER-119) and anti-CD71 (R17217). To assess proliferation, cells were incubated with surface antibodies and annexin V in binding buffer prior to fixation. Cells were fixed using the FoxP3/Transcription Factor Staining Buffer Kit (Invitrogen) according to manufacturer’s instructions. Following fixation cells were stained intracellularly for Ki67 and FoxP3. To assess T follicular cells, staining was performed as previously described using a two-step CXCR5 staining protocol^19^. Flow cytometry was performed on a Becton Dickinson LSR-II and data analyzed using FCS Express with Diva Version 4 (DeNovo Software) or FlowJo.Version 7.6.5 (FlowJo). Antibodies are outlined by staining panel in Supplementary Table S2.

### Intracellular cytokine stains

For evaluation of cytokine production, intracellular cytokine staining was performed as previously described^52,68^. Lymphocytes and splenocytes were stimulated for 5 h at 37 °C with 70 ng/mL phorbol 12-myristate 13-acetate (PMA; Fisher Scientific) and 700 ng/mL ionomycin (MP-Biomedicals) and treated with 5 μg/mL brefeldin A (MP-Biomedicals)^30^. Cells were then surface stained with anti-CD4 (RM4-5), anti-CD8α (53–6.7), and for exclusion of non-relevant cells [anti-CD45R (B220; RA3-6B2), anti-CD11b (MI/70), anti-CD11c (N418), and anti-Ly-6G (Gr-1; RB6-8C5)]. Cells were fixed in BD Cytofix/Cytoperm (BD Biosciences) according to manufacturer’s instructions. Next, cells were incubated for 45 min at 4 °C in 0.5% saponin/PBS with anti-IL-2 (JES6-5H4) and anti-IFNγ (XMG1.2), or IL-10 (JES5-16E3) and CTLA-4 (UC10-4F10-11; BD Pharmigen).

### T cell stimulations and phospho-flow cytometry

For all signaling assays, non-sorted splenocytes or lymph node cells were serum-starved for 1 h at 37 °C before stimulation in complete media without serum. For evaluation of TCR signaling, splenocytes and lymphocytes were stimulated with 20 μg/mL anti-CD3ε (145-2C11; Biolegend) and 50 µg/mL anti-IgG (polyclonal, α-Armenian hamster, Jackson ImmunoResearch) for the indicated times^69,70^. For evaluation of pSTAT5 signaling, cells were stimulated for 15 min with recombinant human IL-2 (rhIL-2; NIH AIDS Reagent Program) at indicated concentrations, recombinant mouse IL-15 (rmIL-15; Peprotech) at indicated concentrations, or 10 ng/mL recombinant IL-7 (rmIL-7; Peprotech). Immediately following stimulation cells were fixed with a final concentration of 1.5% methanol-free formaldehyde (Fisher Scientific; Cat. PI28906) for 15 min, and permeabilized with 500 µL of ice-cold methanol per 1 × 10^6^ cells for 30 min^69,71^. Cells were washed twice with PBS/2%FBS (Omega Scientific) then stained for 60 min in anti-CD4 (RM4-5), anti-CD8α (53-6.7), anti-FoxP3 (FJK-16s), anti-pSTAT5 (Y694; C71E5; Cell Signaling Technologies) or anti-pS6 (S235/236; D57.2.2E; Cell Signaling Technologies), and, for exclusion of non-relevant cells, anti-CD45R (B220; RA3-6B2), anti-CD11b (MI/70), anti-CD11c (N418), and anti-Ly-6G (Gr-1; RB6-8C5).

### Flow cytometry gating strategies

In all analysis, leukocytes were gated on forward and side scatter with doublets excluded, then for viability by exclusion of eFluor506 viability dye. For T cell analysis, non-relevant cells were excluded then CD4 or CD8 T cells were gated for feature characterization. Treg cells were defined as FoxP3+ cells within either CD4+ or CD8+ gates. Hematopoietic cell populations were analyzed by the indicated surface expression following exclusion of lineage positive cells—Lineage-negative, Sca-1-positive, c-Kit-positive progenitors (LSK; CD127^−^ Sca-1^+^ c-Kit^+^), CLP (CD127^+^ Sca-1^int^ c-Kit^int^), megakaryocyte-erythrocyte progenitor (MEP; CD127^−^ Sca-1^−^ c-Kit^+^ CD34^−^ CD16/32^−^), common myeloid progenitor (CMP; CD127^−^ Sca-1^−^ c-Kit^+^ CD34^+^ CD16/32^−^), and granulocyte-monocyte progenitor (GMP; CD127^−^ Sca-1^−^ c-Kit^+^ CD34^+^ CD16/32^+^). RBC precursors in the BM were analyzed by expression of CD71 and Ter119 following exclusion of CD71^−^Ter119^−^ cells – RBC I (CD71^+^ Ter119^−^), RBC II (CD71^+^ Ter119^+^), RBC III (Ter119^+^ CD71^int^), RBC IV (Ter119^+^ CD71^−^)^11,24^. Thymic T cell subsets were gated based on CD4 and CD8 expression following elimination of non-relevant cells based on CD11b, CD11c, CD496, CD45R, and Ly6-G. Double-negative T cells were further divided into subsets 1–4 by CD25 and CD44 expression (1: CD44+CD25−; 2: CD44+CD25+; 3: CD44−CD25+; 4: CD44−CD25−). Combined subsets 1 and 2, and 3 and 4 were defined by expression of CD44 alone.

### Principal component analysis (PCA)

PCA was performed using singular value decomposition in RStudio using R version 3.4.0 through the Bioconductor “pcaMethods” package. PCA was initially performed on 14 parameters acquired from CBC and RBC Ab detection, including frequencies of RBC-bound IgM and RBC-bound IgG, and concentrations of RBC, white blood cells, platelets, neutrophils, hemoglobin, lymphocytes, monocytes, eosinophils, and basophils, hematocrit, mean platelet volume, and mean corpuscular volume. To determine which of these 14 parameters most contributed to separation between early and late disease mice, regression tree analysis via Chi-square automatic interaction detection was performed using XLSTAT-Base Version 19.4.46344 with Bonferroni correction, excluding observations with missing data. This identified four parameters that contributed the most to the variance, and these four parameters—WBC, RBC, hematocrit, and mean platelet volume—were used for subsequent principal component analyses. R script used for 14-variable PCA shown below, with annotation. Script for 4-variable PCA only differs by file name. To evaluate causes of overall disease kinetics, PCA was performed similarly on 35 parameters that were all collected from the same mice, including RBC, hemoglobin, hematocrit, and frequencies of RBC-bound IgM, RBC-bound IgG, LN naïve CD4 T cells, LN CD4 activated/effector memory T cells, LN CD4 central memory T cells, LN CD8 central memory T cells, SPL CD4 T cells, SPL CD8 T cells, SPL CD4:CD8 ratio, SPL naïve CD4 T cells, SPL CD4 activated/effector memory T cells, SPL CD4 central memory T cells, SPL naïve CD8 T cells, SPL CD8 activated/effector memory T cells, SPL CD8 central memory T cells, SPL CD4 T follicular helper, SPL CD8 T follicular cell, BM LSK, BM CLP, BM CMP, BM GMP, BM MEP, and BM RBC precursors 1–4. Following regression tree analysis, four parameters were identified as most useful in separating the data—RBC, RBC-bound IgM, splenic CD4 central memory T cells, and splenic CD8 central memory T cells—which were then used alone for PCA analysis.

### Statistics

All statistics were performed in GraphPad Prism Version 7 or 8 or RStudio with R version 3.4.0. For evaluations with five group comparisons, one-way ANOVA with multiple comparisons test and Benjamini–Hochberg FDR correction was performed. For data with only three group comparisons, student’s t tests were performed. When standard deviations were significantly different between groups, Welch’s correction was applied to the student’s t test. Grubb’s test for outliers was utilized to determine whether significant outliers were present to exclude. Log-rank (Mantel-Cox) test was performed to compare Kaplan–Meier survival curves. Pearson’s correlations were used to assess linear regressions for peripheral blood data. F tests were performed as part of linear regression analyses to determine whether the slopes are significantly non-zero.

## Supplementary Information

Supplementary Information
