# Supplementary material for: T cell signaling and Treg dysfunction correlate to disease kinetics in IL-2Rα-KO autoimmune mice
Source: Sci Rep. 2020 Dec 15;10:21994. doi: 10.1038/s41598-020-78975-y (PMC7738527; doi:10.1038/s41598-020-78975-y)
Supplement: Supplementary file 1 — Supplementary Information [file 41598_2020_78975_MOESM1_ESM.pdf]

T cell signaling and Treg dysfunction correlate to disease kinetics in IL-2Rα-KO autoimmune mice

Genevieve N. Mullins[1,3], Kristen M. Valentine[1,3], Mufadhal Al-Kuhlani[2]
Dan Davini[2], Kirk D.C. Jensen[1,2,3], and Katrina K. Hoyer[1,2,3]

[1]Quantitative and Systems Biology Graduate Program, University of California Merced, Merced, CA 95343
[2]Department of Molecular and Cell Biology, School of Natural Sciences, University of California Merced, Merced, CA 95343
[3] Health Sciences Research Institute, University of California Merced, Merced CA 95343

S1 Table. Mullins et al.

Assessment	Score	Description
Size (based on weight and appearance)	-1	Larger than average for age
	0	Average size for age
	1	Smaller than average for age
Pale tail	1	Noticeably pale tail compared to normal pink appearance
Pale front paws	1	Noticeably pale tail compared to normal pink appearance
Pale rear paws	1	Noticeably pale tail compared to normal pink appearance
Pale ears	1	Noticeably pale tail compared to normal pink appearance
Hunched	1	Hunched appearance seen (generally also lethargic)
Dyspnea	1	Breathing appears difficult (more rapid or shallow than breaths of non-anemic littermates)

S1 Table. Criteria for body scoring of IL-2Rα-KO mice during disease progression.

S2 Table. Mullins et al.

Panel	Antibody (clone)
T cell activation	CD4 (RM4-5), CD8α (53-6.7), CD62L (MEL-14), CD44 (IM7), CD45R (B220; RA3-6B2), CD11b (MI/70), CD11c (N418), Ly-6G (Gr-1; RB6-8C5)
Cytokine Receptor Expression and Tregs	Surface: CD4 (RM4-5), CD8α (53-6.7), CD127 (IL-7Rα; A7R34), CD122 (IL-2Rβ), TCRβ (H57-597)<br>Intracellular: FoxP3 (FJK-16s)
Hematopoietic Progenitor Populations	CD4 (GK1.5), CD8α (53-6.7), CD3ε (145-2C11), CD45R (B220; RA3-6B2), CD11b (MI/70), CD11c (N418), Ly-6G (Gr-1; RB6-8C5), Ter119 (TER-119), CD117 (c-kit; 2B8), Ly6A/E (Sca-1; D7), CD34 (RAM34), CD16/32 (93), CD127 (IL-7Rα; A7R34)
RBC Precursors	Ter119 (TER-119), CD71 (R17217)
Proliferation	Surface: CD4 (RM4-5), CD8α (53-6.7), CD3ε (145-2C11), Annexin V (Invitrogen)<br>Intracellular: Ki67 (SolA15), FoxP3 (FJK-16s)
Follicular T cell	CD4 (RM4-5), CD8α (53-6.7), CD279 (PD-1; J43), CXCR5 (CD185; SPRCL5), CD45R (B220; RA3-6B2), CD11b (MI/70), CD11c (N418), Ly-6G (Gr-1; RB6-8C5)
Thymic subsets	CD4 (RM4-5), CD8α (53-6.7), CD45R (B220; RA3-6B2), CD11b (MI/70), CD11c (N418), Ly-6G (Gr-1; RB6-8C5), CD496 (integrin α2; DX5), CD44 (IM7), CD25 (PC61.5)
Thymic and BM CLP Stain	CD4 (GK1.5), CD8α (53-6.7), CD3ε (145-2C11), CD45R (B220; RA3-6B2), CD11b (MI/70), CD11c (N418), Ly-6G (Gr-1; RB6-8C5), Ter119 (TER-119), CD496 (integrin α2; DX5), CD117 (c-kit; 2B8), Ly6A/E (Sca-1; D7), CD127 (IL-7Rα; A7R34)

S2 Table. Antibodies, including clones, listed for the flow cytometry panels used.

S1 Fig. Mullins et al.

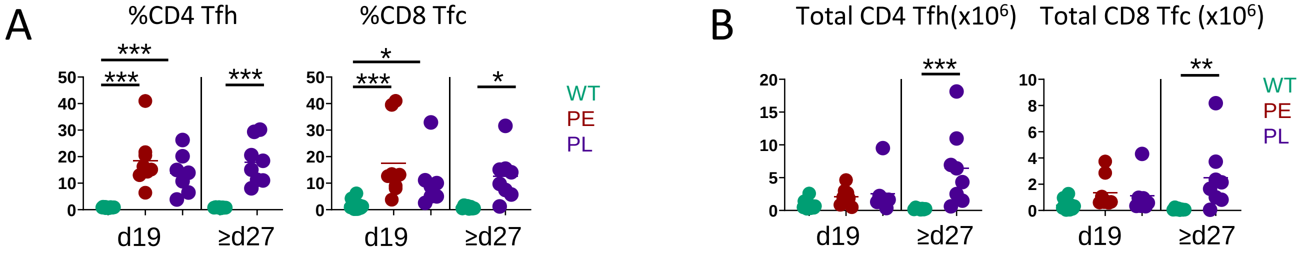

S1 Fig. IL-2Rα-KO mice have increased follicular T cells. (A) Frequency and (B) total number of CD4 T follicular helper (CD4 Tfh) and CD8 T follicular (CD8 Tfc) cells in day 19 and late endpoint (d27+) WT and IL-2Rα-KO spleen are shown. CD4 Tfh defined as CD4+PD-1+CXCR5+; CD8 Tfc defined as CD8+PD-1+CXCR5+. Statistics: one-way ANOVA with Benjamini-Hochberg FDR correction; ** p<0.01, *** p<0.001; *n* = 6-15 mice per experimental group. Non-significant comparisons are not shown. For total number, comparisons between mice of different ages are not shown due to differences in mouse size.

S2 Fig. Mullins et al.

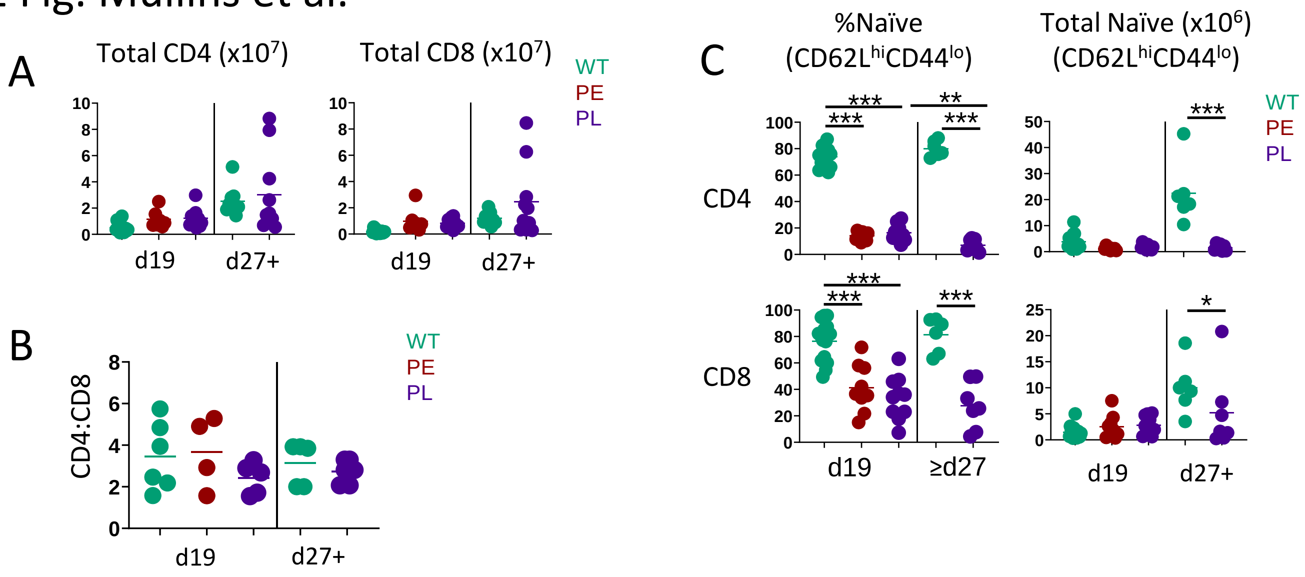

S2 Fig. IL-2Rα-KO T cells express markers indicative of developing memory. (A) Total number of CD4 and CD8 T cells and the ratio of CD4:CD8 T cells in spleen are shown for d19 and late endpoint (d27+) WT and IL-2Rα-KO mice. *n* = 7-14 mice per experimental group. (B) Ratio of single-positive CD4:CD8 T cells in the thymus are shown for d19 and late endpoint (d27+) WT and IL-2Rα-KO mice. *n* = 5-6 mice per experimental group. (C) Summary of frequency and total number of naive CD4 and CD8 T cells in the spleen of d19 and late endpoint (d27+) WT and IL-2Rα-KO mice. *n* = 6-15 mice per experimental group. Statistics: one-way ANOVA with Benjamini-Hochberg FDR correction; * p<0.05, ** p<0.01, *** p<0.001. Non-significant comparisons are not shown. For total number, comparisons between mice of different ages are not shown due to differences in mouse size.

S3 Fig. Mullins et al.

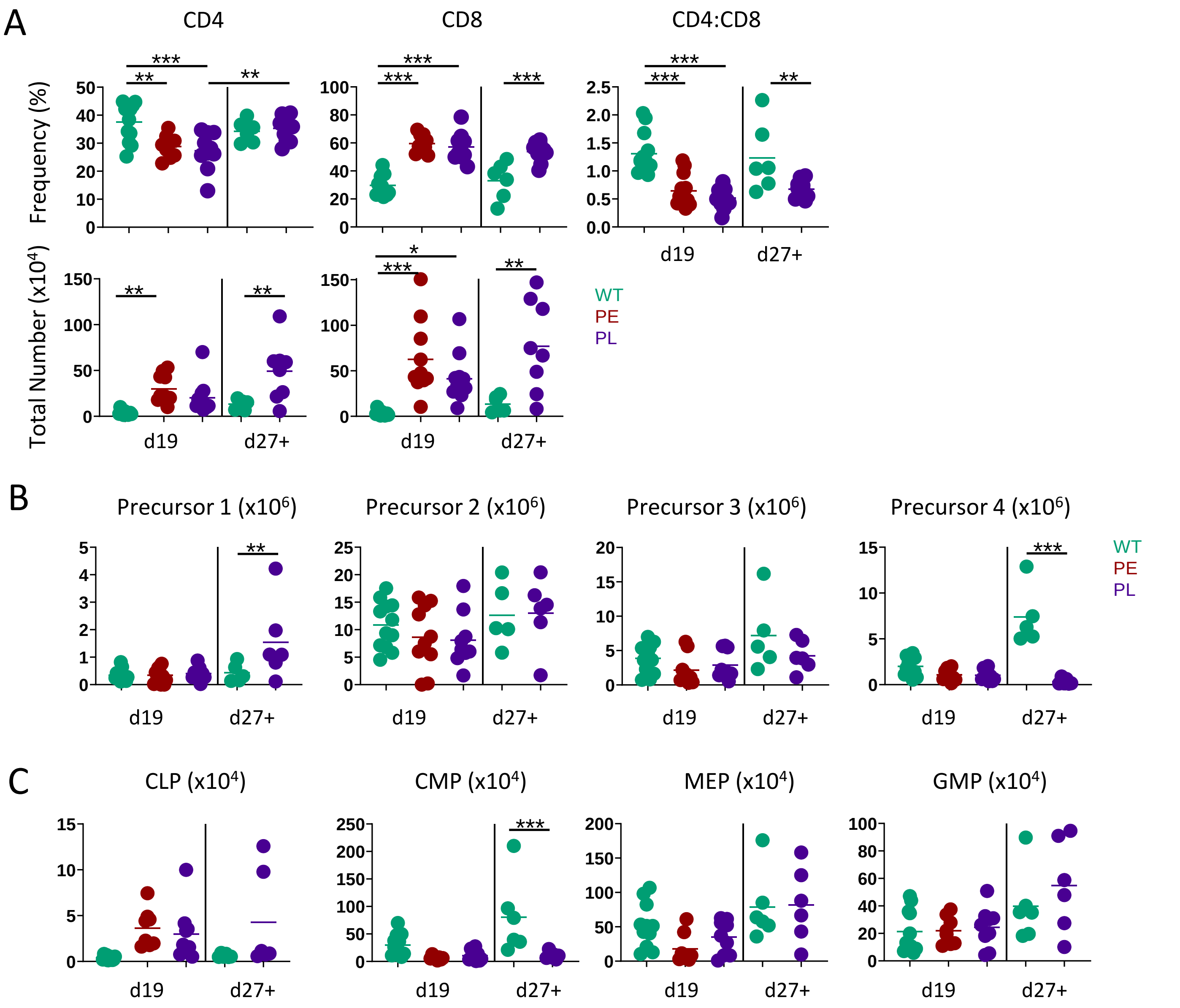

S3 Fig. IL-2Rα-KO mice exhibit kinetic-dependent differences in CLP and RBC precursor frequency. (A) Frequency of TCRβ+ and total number of CD4 and CD8 T cells and the ratio of CD4:CD8 T cells in BM are shown for d19 and late endpoint (d27+) WT and IL-2Rα-KO mice. (B) Total number of RBC precursor populations in d19 and late endpoint (d27+) WT and IL-2Rα-KO mice. (C) Total number of hematopoietic progenitor populations in d19 and late endpoint (d27+) WT and IL-2Rα-KO mice are shown. Statistics: one-way ANOVA with Benjamini-Hochberg FDR correction; * $p<0.05$, ** $p<0.01$, *** $p<0.001$; *n* = 9-12 mice per experimental group. Non-significant comparisons are not shown. For total number, comparisons between mice of different ages are not shown due to differences in mouse size.

S4 Fig. Mullins et al.

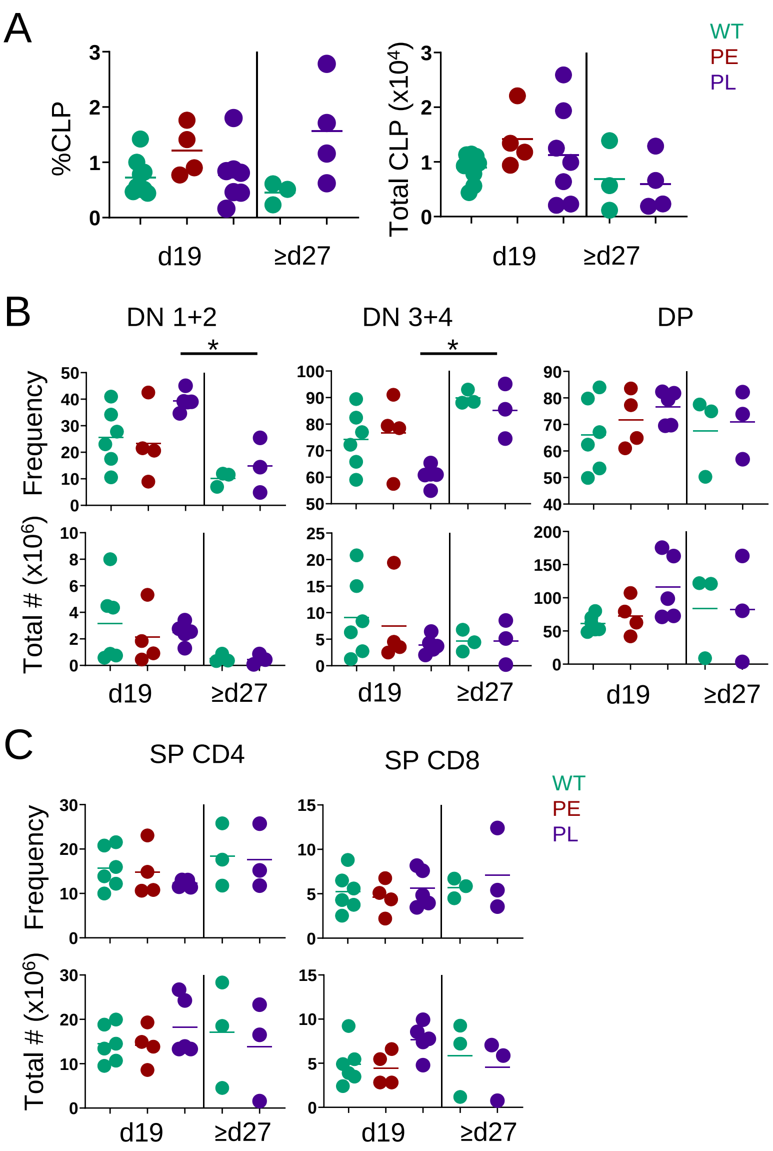

S4 Fig. Thymocyte frequencies are unchanged in IL-2Rα-KO mice. (A) Frequency and total number of CLP in the thymus of d19 and late endpoint (d27+) WT and IL-2Rα-KO mice. (B, C) Frequency and total number of thymic T cell subsets in d19 and late endpoint (d27+) WT and IL-2Rα-KO mice. Abbreviations: single-positive (SP), double-negative (DN), and double-positive (DP). *n* = 3-6 mice per experimental group over 5 independent experiments. Statistics: one-way ANOVA with Benjamini-Hochberg FDR correction; * $p<0.05$, ** $p<0.01$, *** $p<0.001$. Non-significant comparisons are not shown. For total number, comparisons between mice of different ages are not shown due to differences in mouse size.

S5 Fig. Mullins et al.

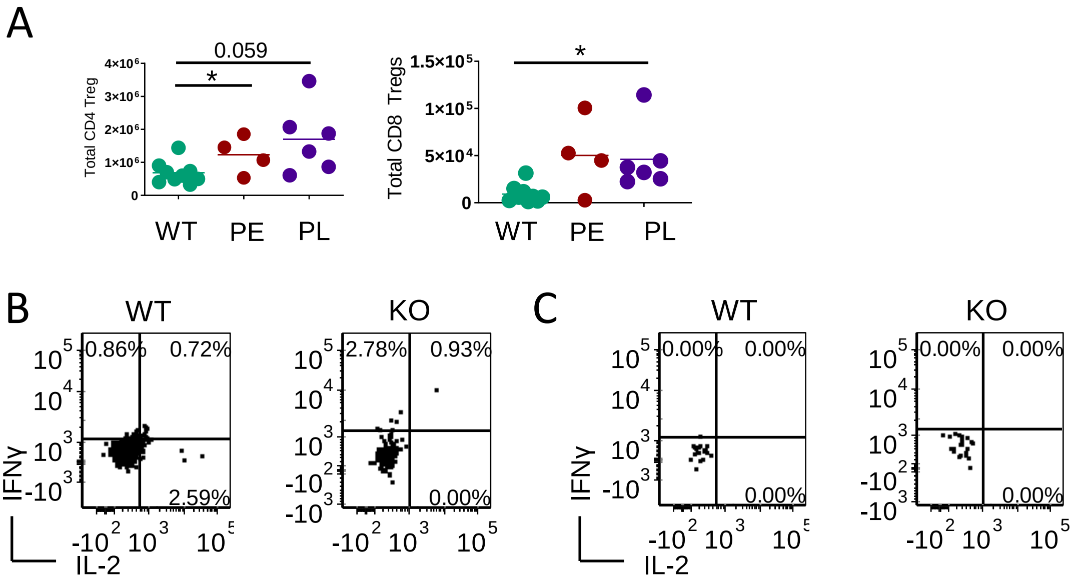

S5 Fig. IL-2Rα-KO CD4 and CD8 Treg cell numbers are comparable to WT. (A) Total number of CD4 and CD8 Tregs in the spleen in d19 WT and IL-2Rα-KO mice. n = 4-10 mice per experimental group. Representative flow plots of IL-2 and IFNγ expression in d19 WT and IL-2Rα-KO (B) CD4 or (C) CD8 Treg cells. Statistics: unpaired Student's t-test. Non-significant comparisons are not shown. For total number, comparisons between mice of different ages are not shown due to differences in mouse size.

S6 Fig. Mullins et al.

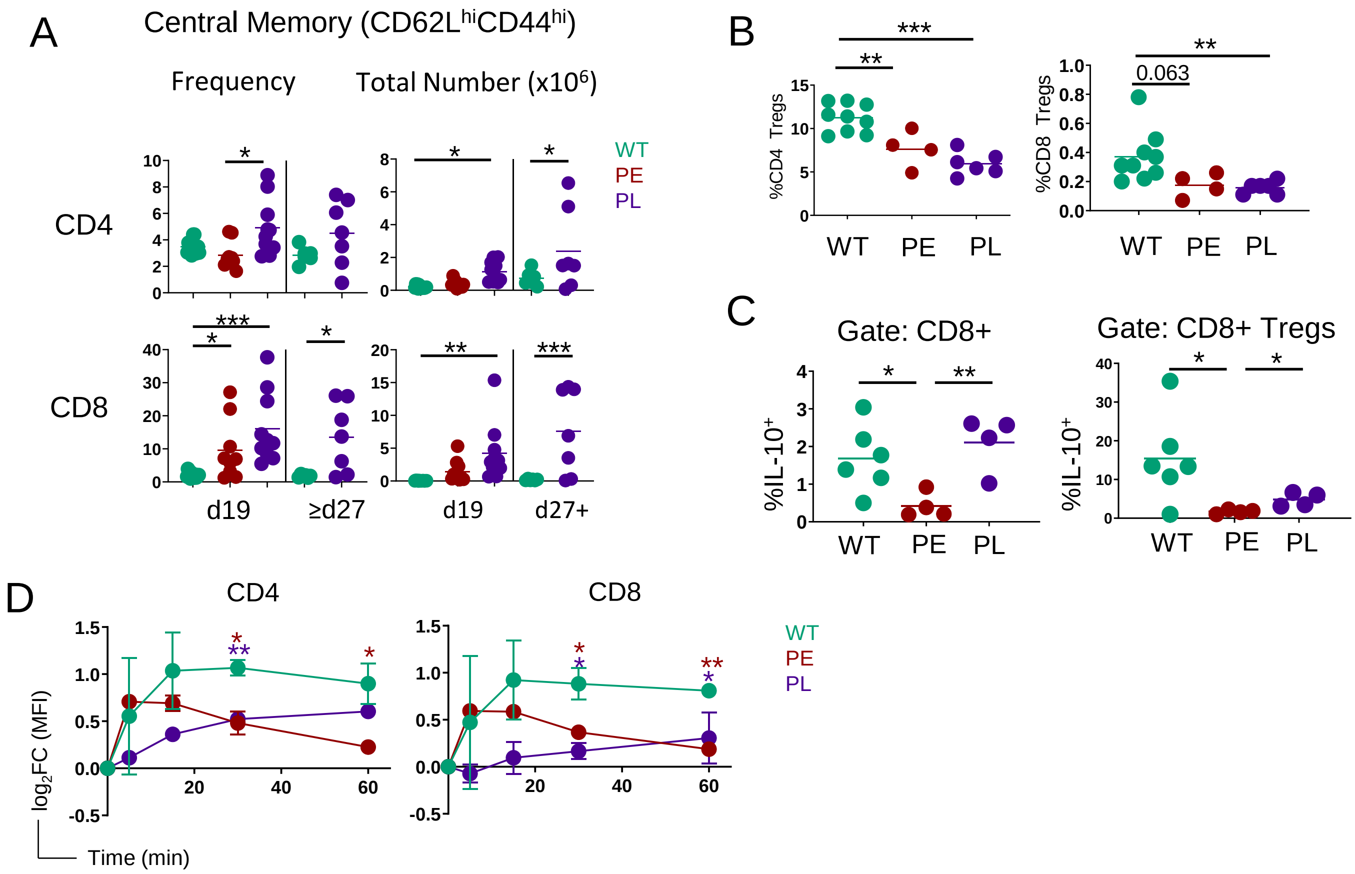

S6 Fig. Lymph node T cells show differences in activation and Treg phenotype. (A) Frequency and total number of lymph node CD4 and CD8 T cell central memory in 19-day-old and late endpoint (d27+) WT and IL-2Rα-KO mice. Statistics: one-way ANOVA with Benjamini-Hochberg FDR correction; *n* = 5-10 mice per experimental group. (B) Frequency of CD4 and CD8 Tregs in the lymph node in 19-day-old WT and IL-2Rα-KO mice. *n* = 4-11 mice per experimental group. (C) Frequency of IL-10+ 19-day-old WT and IL-2Rα-KO lymph node CD8 T and CD8 Treg cells post 5-hour PMA and ionomycin stimulation. *n* = 4-6 mice per experimental group over 4 independent experiments. (D) Log_2 fold change of S6 phosphorylation by mean fluorescent intensity (MFI) from unstimulated over time following TCR stimulation comparing WT versus IL-2Rα-KO mice for lymph node CD4 and CD8 T cells. *n* = 2-3 mice per experimental group from 3 independent experiments. Statistics in (D) are compared to WT and colored by which IL-2Rα-KO group is being compared. Statistics: unpaired Student's t-test. * p<0.05, ** p<0.01, *** p<0.001. Non-significant comparisons are not shown.
